# Analysis of Tumor-Associated Macrophages (TAM) Using Cluster of Differentiation 68 (CD68) Immunohistochemical Marker in Endometrial Carcinoma

**DOI:** 10.7759/cureus.109982

**Published:** 2026-05-31

**Authors:** Kiruthiga K, Subalakshmi Balasubramanian, Sandhya Sundaram

**Affiliations:** 1 Pathology, Sri Ramachandra Institute of Higher Education and Research, Chennai, IND

**Keywords:** cd68, cluster of differentiation, endometrial carcinoma, immunohistochemical marker, tumor-associated macrophages

## Abstract

Introduction

Endometrial carcinoma is a common gynaecological malignancy in which tumor progression is influenced not only by neoplastic cells but also by the tumor microenvironment. Tumor-associated macrophages (TAMs) play a key role in modulating tumor behavior and provide prognostic insights depending on their distribution pattern.

Objectives

To evaluate Tumor-associated macrophages (TAMs) using CD68 immunohistochemistry marker and analyze their spatial distribution in endometrial carcinoma, and to correlate the localization of TAM with tumor grade, stage, myometrial invasion, and lymph node metastasis.

Materials and methods

This retrospective pilot study was conducted in the Department of Pathology at Sri Ramachandra Medical College and Research Institute from January 2024 to December 2024, using seven formalin-fixed paraffin-embedded tissue samples of histopathologically confirmed endometrial carcinoma. Tissue sections were stained with the CD68 antibody, which highlights TAMs. Their distribution was assessed as stromal, intratumorally, and along the invasive margin. Clinicopathological data were retrieved from lab reports, and the findings were analyzed using Statistical Package for Social Sciences (SPSS), version 16 (SPSS Inc., Chicago, IL)

Results

The mean age of study participants was 63.0 ± 7.28 years, with endometrioid carcinoma being the most common subtype, five cases (71.4%). CD68-positive TAMs showed a heterogeneous distribution, located in the stroma in four cases (57.1%), intratumorally in two cases (28.6%), and at the invasive margin in one case (14.3%). There was no statistically significant association observed between TAM localization and tumor grade (p=1.00) or stage (p=1.00).

Conclusion

TAMs in endometrial carcinoma demonstrate spatial heterogeneity with stromal predominance, suggesting a role in tumor microenvironment modulation. However, CD68-based assessment alone showed no significant association with clinicopathological parameters in our study. Hence, further studies with a larger sample size and additional markers are recommended.

## Introduction

Endometrial carcinoma is one of the most common malignancies of the female genital tract, considered a growing public health problem due to its rising incidence, contributing significantly to cancer-related morbidity and mortality worldwide. The progression of endometrial carcinoma is not solely dependent on the presence of malignant epithelial cells but also profoundly influenced by the microenvironment of the tumor, composed of stromal cells, immune cells, cytokines, and extracellular matrix components [1,2].

Tumor-associated macrophages (TAMs) originate from circulating monocytes. They exhibit plasticity, enabling monocytes to adopt diverse functional phenotypes in response to microenvironmental signals. TaMs displays a spectrum towards an anti-inflammatory M2-like phenotype, which promotes tumor progression. These cells facilitate angiogenesis, cellular proliferation, and immune evasion through the secretion of growth factors, cytokines, and immunosuppressive mediators [3].

The importance of TAM has been a topic of interest in carcinomas of the breast, cervix, prostate, renal, and esophagus. TAM infiltration is negatively associated with clinicopathological features in endometrial cancer, including deep myometrial invasion, advanced stage, and poor prognosis [4]. The complexity of tumor-immune interactions becomes clear with the metabolic links between tumor cells and macrophages. For example, lactate signaling encourages macrophages to adopt behaviors that support tumors. These findings emphasize the potential of TAMs as treatment targets and as a marker for prognosis [5].

An immunohistochemical analysis is an important step in determining and quantifying tumor-associated macrophages in tumor tissues [6]. Cluster of Differentiation 68 (CD68) is one of the markers that is used widely for determining macrophages in histopathological specimens. CD68-positive macrophages have been observed in tumors as well as in tissues surrounding tumors, and help researchers in the quantification of these macrophages. It is important from an analytical perspective in order to determine the number of macrophages present within tumor tissues and also their correlation with the aggressiveness of tumors. As these tumor-associated macrophages play an important role in modulating tumor behavior, this study aims to assess these macrophages within endometrial carcinomas using the CD68 marker [7].

## Materials and methods

We performed a retrospective pilot study in the Department of Pathology at Sri Ramachandra Medical College and Research Institute from January 2024 to December 2024. Seven archived samples of formalin-fixed tissue blocks of pathologically proven endometrial carcinoma were included. Poorly preserved, inadequate histopathological specimens and cases lacking a definitive histopathological diagnosis were excluded from the study. The study was conducted after obtaining approval from the Institutional Ethical Committee, Sri Ramachandra Medical College and Research Institute (CSP-MED/26/MAR/126/82).

Sections of 4 μm thickness were mounted on charged slides, dried overnight at room temperature, and incubated at 60 degree celsius for an hour. Deparaffinisation was done, followed by epitope retrieval by the pressure cooker method using Tris ethylene diaminetetracetic acid (EDTA) buffer. A ready-to-use antibody was added to the slides. Tissue binder and horseradish peroxidase were then added. DAB chromogen was used to highlight the positivity, followed by counterstaining with hematoxylin and mounting. Sections were examined under a light microscope, and representative areas where TAMs accumulated at greater density were identified. Clinicopathological characteristics such as age, sex, tumor size, histological type, histological grade, and stage, lymph node and distant metastatic status were collected from the laboratory information system. Data were entered into Microsoft Excel (Microsoft, Redmond, WA) and analyzed using Statistical Package for Social Sciences (SPSS) version 16 (SPSS Inc., Chicago, IL). Descriptive statistics were used to summarize the clinicopathological characteristics of the study population. Continuous variables, such as age, were expressed as mean ± standard deviation, while categorical variables, such as histological type, grade, tumor size, depth of myometrial invasion, lymph node status, and CD68 expression patterns, were presented as frequencies and percentages.

The distribution of CD68-positive TAMs was assessed across different compartments of the tumor microenvironment, namely stromal, intratumoral, and along the invasive margin. Associations between the localization of CD68-positive TAMs and clinicopathological parameters were analyzed using Fisher’s exact test. A p-value of <0.05 was considered statistically significant. The results of the study were interpreted descriptively because of the small sample size. 

## Results

The mean age of study participants was 63.0 ± 7.28 years. The most common histological type of endometrial carcinoma observed was endometrioid endometrial carcinoma in five cases (71.4%), of which one was a synchronous tumor with ovarian carcinoma. Serous carcinoma was a less common subtype, observed in two cases (28.6%). International Federation of Gynecology and Obstetrics (FIGO) Grade 1 was reported in five cases (71.4%), Grade 2 in one case (14.3%), and Grade 3 in one case (14.3%). Myometrial invasion was observed in all the cases. Only one case (14.3%) had lymphovascular invasion. 

The tumor was staged according to the 2025 College of American Pathologists (CAP) guidelines. Six cases (85.7%) were staged as T1 and one case (14.3%) as T2. According to FIGO (2023) classification, four cases (57.4%) were staged under FIGO IA2, one case (14.3%) under FIGO IIA, one case (14.3%) under FIGO IIB, and one case (14.3%) under FIGO IIC. Lymph node metastasis was identified in one case (14.3%) (Table 1).

**Table 1:** Clinicopathological characteristics of study participants (n=7) FIGO: International Federation of Gynecology and Obstetrics. Data are presented as numbers (n) and percentages (%).

Variable	Frequency (%)
Age	
<60 years	1 (14.3%)
>60 years	6 (85.7%)
Histological types of endometrial carcinoma	
Endometrioid carcinoma	5 (71.4%)
Serous carcinoma	2(28.6%)
FIGO Grade	
Grade 1	5 (71.4%)
Grade 2	1 (14.3%)
Grade 3	1 (14.3%)
Lymphovascular invasion	
Present	1 (14.3%)
Absent	6 (85.7%)
Lymph node metastasis	
Present	1 (14.3%)
Absent	6 (85.7%)
pTNM Staging	
pT1	6 (85.7%)
pT2	1 (14.3%)
FIGO Staging	
Stage IA2	4 (57.1%)
Stage IIA	1 (14.3%)
Stage IIB	1 (14.3%)
Stage IIC	1 (14.3%)

**Figure 1:**
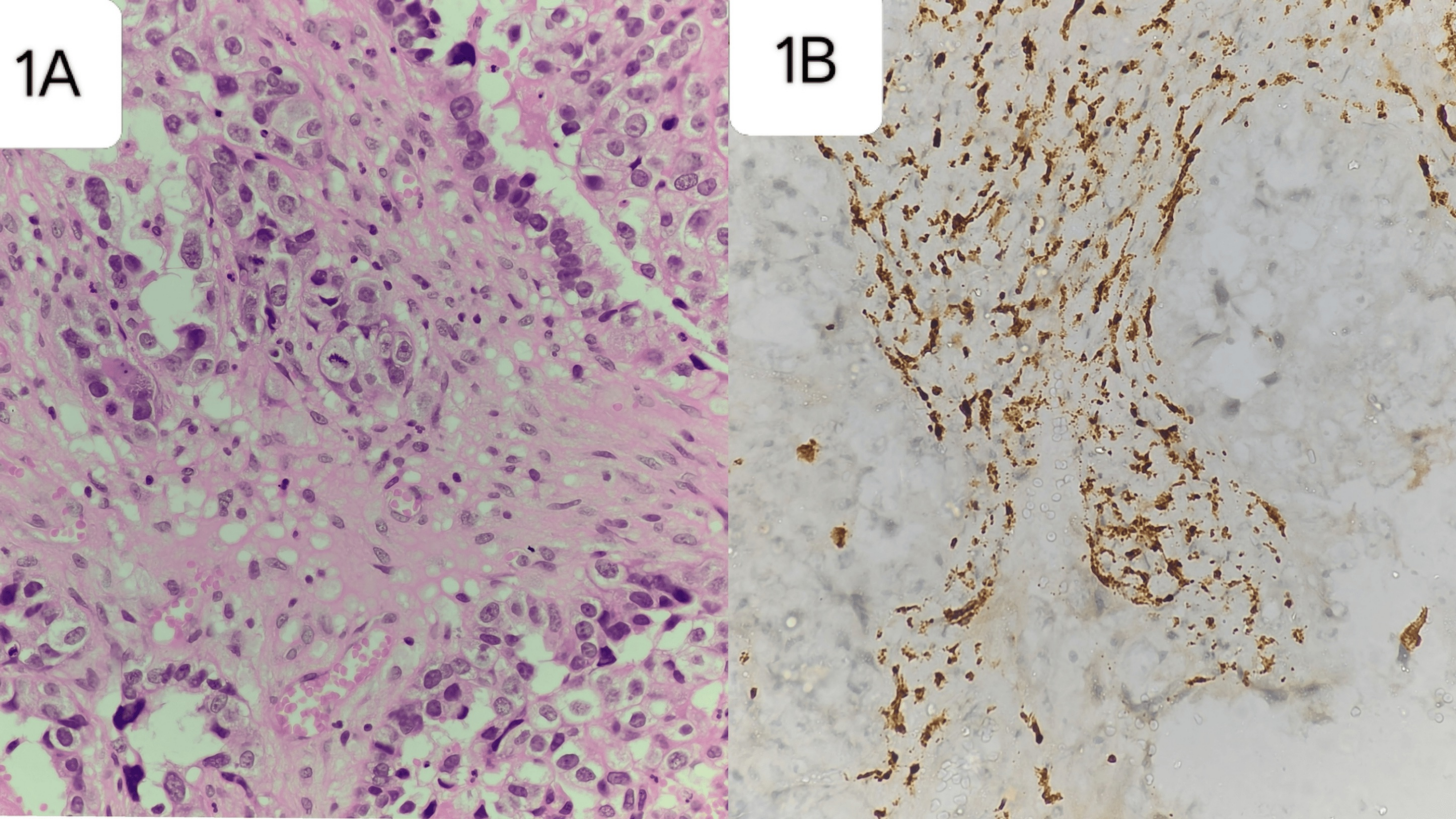
Immunohistochemistry result images (1) (A) Photomicrograph of Hematoxylin and Eosin stain showing TAM in the stroma at 400X magnification. (B) Photomicrograph of immunohistochemistry (IHC) for CD68 marker highlighting Stromal TAMs at 400X magnification.

**Figure 2:**
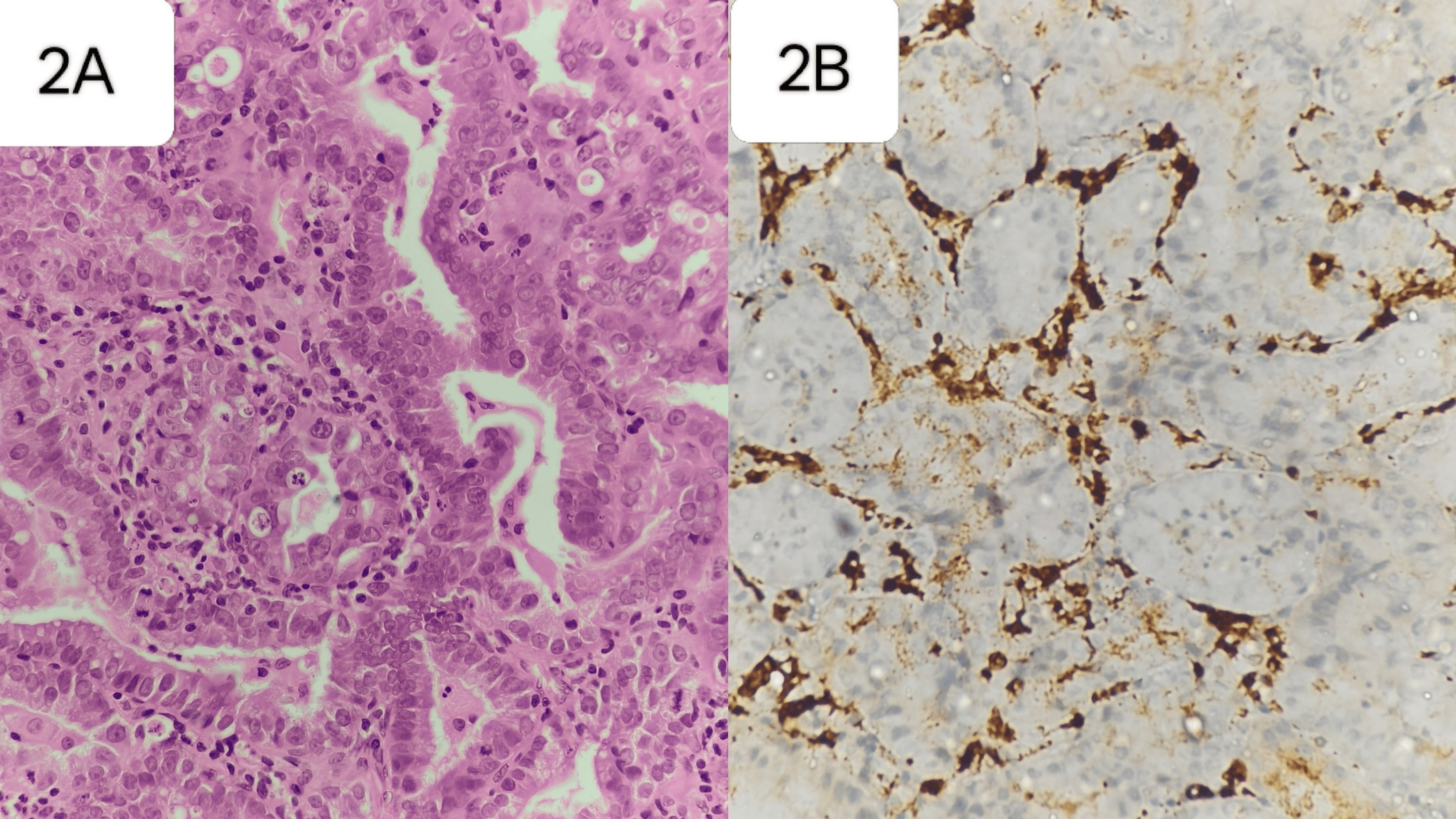
Immunohistochemistry result images (2) (A) Photomicrograph of Hematoxylin and Eosin stain showing TAM within the tumor at 400X magnification. (B) Photomicrograph of immunochemistry (IHC) for CD68 marker highlighting Intratumorally located TAM at 400X magnification.

**Figure 3:**
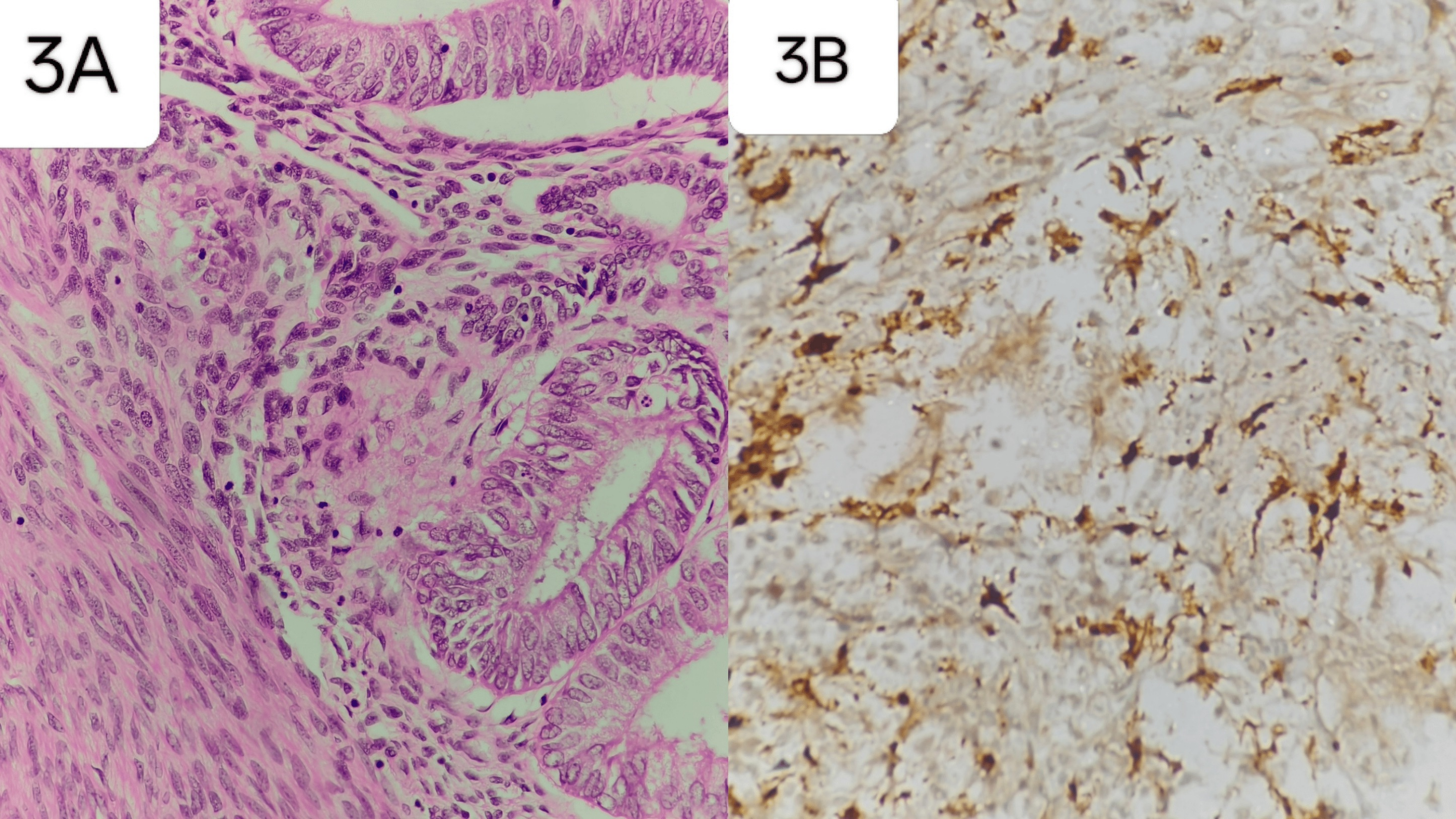
Immunohistochemistry result images (3) (A) Photomicrograph of Hematoxylin and Eosin stain showing TAMs at the invasive margin at 400X magnification. (B) Photomicrograph of immunohistochemistry (IHC) for CD68 marker highlighting TAMs at the invasive margin at 400X magnification.

TAM was highlighted in all the cases. The immunohistochemical distribution of CD68-positive macrophages was observed in the stroma in four cases (57.1%), intratumorally in two cases (28.6%), and at the invasive margin in one case (14.3%), which highlighted spatial heterogeneity (Figures 1-3) (Table 2).

**Table 2:** Distribution of CD68 expression in tumor microenvironment CD68: Cluster of Differentiation, TAM: Tumor-associated macrophages.

Location of CD68-highlighted TAM	Frequency (%)
Stromal	4 (57.1%)
Intra tumoral	2 (28.6%)
Invasive margin	1 (14.3%)

Among the five cases of endometrioid endometrial carcinoma, stromal distribution of TAMs was seen in three cases (60%), intratumoral TAMs in one case (20%), and TAMs at the invasive margin in the other case (20%) showed TAMs at the invasive margin. Of the two serous types, one case (50%) had intratumoral TAM and the other (50%) had TAM at the invasive margin (Table 3).

**Table 3:** Association of CD68 expression with tumor type CD68: Cluster of Differentiation 68. Data are presented as numbers (n) and percentages (%).

Type	Stromal	Non-stromal	p-value
Endometrioid type	3 (60%)	2 (40%)	1.00
Serous type	1 (50%)	1 (50%)	

Stromal predominance was observed across all FIGO tumor grades. One case (14.3%) showed TAM at the invasive margin. Among six cases of pT1 tumors, the stromal pattern of distribution dominated in four cases (66.6%) in comparison to other patterns. One case with pT2 had TAM at the invasive margin (Table 4).

**Table 4:** Association of CD68 expression with tumor grade (n=7) CD68: Cluster of Differentiation 68. Data are presented as numbers (n) and percentages (%).

Grade	Stromal	Non-stromal	p-value
Low grade	3 (50%)	3 (50%)	1.00
High grade	1 (100%)	0	

In five cases of FIGO Stage I tumors, three cases demonstrated a stromal pattern, one within the tumor and one along the invasive margin. In two cases of FIGO Stage II, one case had a stromal pattern, and the other had TAM within the tumor. TAM was seen along the invasive margin in one case with lymph node metastasis (Table 5).

**Table 5:** Association of CD68 Expression with tumor FIGO stage FIGO: International Federation of Gynecology and Obstetrics, CD68: Cluster of Differentiation 68. Data are presented as numbers (n) and percentages (%).

Stage	Stromal	Non-stromal	p-value
Stage I	3 (60%)	2 (40%)	1.00
Stage II	1 (50%)	1 (50%)	

The majority of CD68-positive TAMs were localized to the stroma, suggesting a potential role in tumor microenvironment modulation rather than direct tumor cell interaction. There was no statistically significant association between different locations of CD68-positive cells and the clinicopathological characteristics of study participants due to the smaller number of samples.

## Discussion

The primary aim of our study was to identify TAMs using the CD68 IHC marker and analyse their correlation with endometrial cancer. The mean age of the study population in our study was found to be 63.0 ± 7.28 years, which is consistent with the epidemiological profile of endometrial carcinoma occurring predominantly in postmenopausal women [8]. The predominance of low-grade endometrioid histology in our cohort reflects the usual pattern of endometrial cancer presentation [9], particularly in cases detected at earlier stages or in tumors with relatively favourable biology.

CD68-positive TAMs were found mainly in the stroma, with fewer cells localized intratumorally and along the invasive margin. This stromal predominance suggests that macrophages in endometrial carcinoma may act primarily by shaping the tumor microenvironment through paracrine signaling, immune regulation, angiogenesis, and matrix remodeling rather than by direct interaction with tumor nests. A study by Matsukowa et al. on spatial compartmentalization of macrophages in endometrial cancer and other malignancies supports the concept that macrophage localisation may be as important as macrophage density in understanding the behaviour of a tumor [10].

The predominance of stromal CD68-positive macrophages in our study is biologically plausible, as the stromal compartment is the main site of tumor-host interaction. Stromal macrophages may promote tumor progression by secreting cytokines, growth factors, and proteolytic enzymes that influence invasion and vascular remodeling. The predominance of stromal TAMs suggests that tumor nature is mediated by the microenvironment rather than by direct contact with tumor cells, as Bingle et al. underscored that TAMs in the stroma can drive tumor progression by creating a supportive niche for malignant cells [11]. Low-grade tumors showed the majority of TAM in the stromal region. Kubler et al., in a study on TAM in endometrial carcinoma and its prognostic significance, mentioned that proliferation of new lymphatic channels is stimulated by TAM and potentiates the lymphatic spread of tumour [12]. An association between high TAM density and lymph node metastasis has been observed. 

The presence of TAM in tumors of early stage also showed stromal predominance. Zinovkin et al., in their study on the M1 and M2 subtypes of macrophages in the tumor microenvironment, found that reduced TAMs in the stromal region around the tumor are associated with worse prognosis [13]. This is due to the reduction in the anti-tumor effect of M1. Soeda et al.'s study in endometrioid endometrial carcinoma found that macrophages along the invasive margin were significantly associated with FIGO stage, histological grade, myometrial invasion, lymph node metastasis, and vascular space invasion, while intratumoral macrophages correlated with proliferative and angiogenic markers [14]. Nagamine et al. in their study on intratumoral macrophages in endometrial carcinoma reported that a higher density of TAMs, particularly the M1 phenotype, was found in the cured group, highlighting the importance of TAM density in anti-tumor effects [15]. Although our study did not demonstrate statistically significant associations, the pattern of stromal predominance supports the same underlying biological framework. 

In contrast to studies that reported significant clinicopathological associations, our analysis did not show a statistically significant relationship between CD68 localization and other clinicopathological variables. This may be explained primarily by the very small sample size. With only seven cases overall and even fewer cases available for subgroup analyses, the study was underpowered to detect large effects. The p-values observed in our data, such as 1.00 for grade and 1.00 for stage, suggest a possible trend that may become meaningful in a larger cohort study, as Zhang et al.'s study [16], which showed that TAM density in distinct tumor compartments correlates with patient outcomes. This is an important point to stress because non-significant findings in small pilot studies should not be interpreted as evidence of no biological relationship.

Our findings also align with a study by Krishnan et al. on the spatial distribution of TAMs in endometrial cancer, which reported that CD68-positive macrophages at the tumor margin had prognostic relevance and were associated with clinicopathological outcomes, highlighting that macrophage location [3]. Similarly, a study by Dimitrova et al. evaluated intratumoral and stromal CD68 infiltration and found no significant association with FIGO stage or lymph node status, which is broadly consistent with the lack of significant association observed in our cohort [17]. Altogether, these studies suggest that macrophages are clearly involved in endometrial cancer, and their impact may depend on tumor subtype, disease stage, polarization status, and anatomic compartment.

It is also important to note that CD68 is a general macrophage marker and does not distinguish between functionally distinct macrophage phenotypes. Therefore, CD68 positivity alone cannot determine whether the macrophages are predominantly tumor-promoting or tumor-suppressing. A study by Nesina et al. on endometrial carcinoma suggests that macrophage polarization markers such as CD163 may be more closely linked to aggressive behaviour than CD68 alone, especially when combined with measures of stromal remodelling and angiogenesis [18]. In this context, our findings may reflect the presence of macrophages in the stroma without fully capturing their functional heterogeneity.

The observed predominance of low-grade endometrioid tumors in our study may also have influenced the results. Lower-grade tumors and early-stage lesions may show less pronounced immune remodeling than high-grade or advanced-stage tumors, potentially reducing the ability to detect a strong association between CD68 distribution and adverse clinicopathological variables. Despite these limitations, the present study provides useful preliminary evidence that CD68-positive macrophages are not uniformly distributed in endometrial carcinoma and are concentrated mainly in the stroma. This finding reinforces the concept that TAMs in the tumor microenvironment play a central role in endometrial cancer progression [19] . 

Limitations

A smaller sample size (n = 7) is the primary limitation of our study, which substantially limits the statistical power of the study, so that the findings of the study should be interpreted as exploratory and for hypothesis generation. The study population predominantly consisted of low-grade endometrioid carcinoma, which limits representation of diverse histological subtypes and higher-grade tumors, which may exhibit different immune microenvironment characteristics, thereby limiting generalizability of the results across the spectrum of endometrial carcinoma. Use of a single pan-macrophage marker (CD68) for immunohistochemical assessment does not distinguish among different phenotypes or delineate the relative contributions of tumor-promoting versus tumor-suppressive subsets of endometrial carcinoma. Though the observed stromal predominance emphasized tumor-immune interactions, it has underscored the need for more comprehensive immunophenotypic characterization.

Recommendations

Prospective studies correlating TAM characteristics with patient survival, recurrence, and treatment response to establish prognostic significance. Molecular profiling and treatment based on the profile, as prognostic and predictive tools, are being extensively studied. Adding TAM alongside the above markers and studying it in a larger number of patient samples will provide greater clarity on the matter. Worth further studies in this direction. Future research to evaluate the potential of macrophage-targeted therapies as a treatment strategy for endometrial carcinoma.

## Conclusions

Our retrospective pilot study evaluated the spatial distribution of CD68-positive Tumor Associated Macrophages (TAMs) in endometrial carcinoma and explored their relationship with clinicopathological parameters. CD68-positive TAMs were identified in all three tumor compartments-stromal, intratumorally and at the invasive margin and showed a predominance within the stromal compartment. This pattern supports the concept that macrophages in endometrial carcinoma may influence tumor behavior primarily through modulation of the tumor microenvironment by angiogenesis, immune regulation, and matrix re-modelling in endometrial cancer.

Although no statistically significant association was demonstrated between the compartmental localization of CD68-positive TAMs and tumor grade, size, depth of myometrial invasion, or lymph node metastasis in this cohort, the absence of significance is likely related to the very small sample size and the predominance of low-grade endometrioid tumors, which limited statistical power and histological diversity. Prior studies have shown that TAM density and localization, particularly at the tumor margin and within the stroma, can correlate with more aggressive clinicopathological features and adverse outcomes in endometrial carcinoma, suggesting that macrophage topography may have prognostic relevance in larger, more heterogeneous cohorts.

## References

[1] Sun Y, Jiang G, Wu Q, Ye L, Li B (2023). The role of tumor-associated macrophages in the progression, prognosis and treatment of endometrial cancer. Front Oncol.

[2] Wang Y, Liu N, Guo X, Han R, Bai J, Zhong J, Wang Q (2025). The immune microenvironment in endometrial carcinoma: mechanisms and therapeutic targeting. Front Immunol.

[3] Krishnan V, Schaar B, Tallapragada S, Dorigo O (2018). Tumor associated macrophages in gynecologic cancers. Gynecol Oncol.

[4] Gu X, Shi Y, Dong M, Jiang L, Yang J, Liu Z (2021). Exosomal transfer of tumor-associated macrophage-derived hsa_circ_0001610 reduces radiosensitivity in endometrial cancer. Cell Death Dis.

[5] Liu X, Sun H, Liang J (2025). Metabolic interplay between endometrial cancer and tumor-associated macrophages: lactate-induced M2 polarization enhances tumor progression. J Transl Med.

[6] Jayasingam SD, Citartan M, Thang TH, Mat Zin AA, Ang KC, Ch'ng ES (2019). Evaluating the polarization of tumor-associated macrophages into M1 and M2 phenotypes in human cancer tissue: technicalities and challenges in routine clinical practice. Front Oncol.

[7] Yang Y, Sun D, Ma X, Wang T, Wu J (2025). CD68- and CD163-positive tumor-associated macrophages in renal clear cell carcinoma. Sci Rep.

[8] Forte M, Cecere SC, Di Napoli M (2024). Endometrial cancer in the elderly: characteristics, prognostic and risk factors, and treatment options. Crit Rev Oncol Hematol.

[9] Lucas E, Mills A, Carrick K (2024). Endometrial carcinoma: low-grade endometrioid carcinoma. Gynecologic and Obstetric Pathology 2024 Feb 12 (pp. 1-70). Singapore: Springer Nature Singapore.

[10] Matsukawa T, Yoshikawa N, Liu W (2024). Spatial distribution of tumor-resident macrophages as predictive biomarkers in endometrial cancer. J Obstet Gynaecol Res.

[11] Bingle L, Brown NJ, Lewis CE (2002). The role of tumour-associated macrophages in tumour progression: implications for new anticancer therapies. J Pathol.

[12] Kübler K, Ayub TH, Weber SK (2014). Prognostic significance of tumor-associated macrophages in endometrial adenocarcinoma. Gynecol Oncol.

[13] Zinovkin DA, Pranjol MZ, Bilsky IA, Zmushko VA (2018). Tumor-associated T-lymphocytes and macrophages are decreased in endometrioid endometrial carcinoma with MELF-pattern stromal changes. Cancer Microenviron.

[14] Soeda S, Nakamura N, Ozeki T (2008). Tumor-associated macrophages correlate with vascular space invasion and myometrial invasion in endometrial carcinoma. Gynecol Oncol.

[15] Nagamine M, Ogi H, Hirai M (2025). Low density of intratumoral M1-macrophage infiltration may correlate with worse prognosis in low-grade early-stage uterine endometrioid carcinoma. Gynecol Oncol Rep.

[16] Cao L, Meng X, Zhang Z, Liu Z, He Y (2024). Macrophage heterogeneity and its interactions with stromal cells in tumour microenvironment. Cell Biosci.

[17] Dimitrova P, Vasileva-Slaveva M, Shivarov V, Hasan I, Yordanov A (2023). Infiltration by intratumor and stromal CD8 and CD68 in cervical cancer. Medicina (Kaunas).

[18] Iurchenko NP, Nesina IP, Glushchenko NМ, Buchynska LG (2023). Role of stromal microenvironment in the formation of invasive, angiogenic, and metastatic potential of endometrioid carcinoma of endometrium. Exp Oncol.

[19] Le Saux O, Sabatier R, Treilleux I (2025). Immune landscape and TAM density in endometrial cancer: implications for immune checkpoint inhibitors efficacy. Ther Adv Med Oncol.

